# Chronic mild and acute severe glaucomatous neurodegeneration derived from silicone oil-induced ocular hypertension

**DOI:** 10.1038/s41598-021-88690-x

**Published:** 2021-04-27

**Authors:** Fang Fang, Jie Zhang, Pei Zhuang, Pingting Liu, Liang Li, Haoliang Huang, Hannah C. Webber, Yangfan Xu, Liang Liu, Roopa Dalal, Yang Sun, Yang Hu

**Affiliations:** 1 Department of Ophthalmology, Stanford University School of Medicine, Palo Alto, CA 94304 USA; 2 Department of Ophthalmology, The Second Xiangya Hospital, Central South University, Changsha, 410011 China; 3 Department of Ophthalmology, Union Hospital, Tongji Medical College, Huazhong University of Science and Technology, Wuhan, 430022 China

**Keywords:** Ocular hypertension, Optic nerve diseases

## Abstract

Recently, we established silicone oil-induced ocular hypertension (SOHU) mouse model with significant glaucomatous neurodegeneration. Here we characterize two additional variations of this model that simulate two distinct glaucoma types. The first is a chronic model produced by high frequency (HF) pupillary dilation after SO-induced pupillary block, which shows sustained moderate IOP elevation and corresponding slow, mild glaucomatous neurodegeneration. We also demonstrate that although SO removal quickly returns IOP to normal, the glaucomatous neurodegeneration continues to advance to a similar degree as in the HF group without SO removal. The second, an acute model created by no pupillary dilation (ND), shows a greatly elevated IOP and severe inner retina degeneration at an early time point. Therefore, by a straightforward dilation scheme, we extend our original SOHU model to recapitulate phenotypes of two major glaucoma forms, which will be invaluable for selecting neuroprotectants and elucidating their molecular mechanisms.

## Introduction

Glaucoma is an optic neuropathy characterized by peripheral to central loss of visual field due to progressive degeneration of optic nerve (ON) and retrograde death of retinal ganglion cells (RGCs)^1–9^. In addition to age and ancestry, intraocular pressure (IOP) is another important risk factor for glaucoma^8^. Although glaucoma can occur at any IOP level if untreated^10^, elevated IOP is associated with accelerated progression, probably due to the sensitivity of the ON head to IOP^5,11,12^. Lowering IOP is the only available treatment but fails to completely prevent the progression of glaucomatous neurodegeneration^13–16^. Deciphering the molecular mechanisms of glaucomatous degeneration, and testing therapeutic targets for neuroprotection and regeneration, require reliable and clinically relevant glaucoma animal models. Rodents have been used extensively to model glaucoma because their anatomical structures and aqueous humor dynamics are similar to those of the human eye^17^; mouse has been particularly useful because of its well-developed transgenic techniques.

Almost all of the previous inducible glaucoma models increase IOP by decreasing aqueous outflow, either through occluding the angle of the anterior chamber or by damaging the trabecular meshwork (TM)^18,19^. However, IOP is maintained by a balance between production and outflow of aqueous humor. Aqueous humor is constantly produced by the ciliary body in the posterior chamber, flows into the anterior chamber through the pupil and out through the TM at the angle of the anterior chamber. Rather than obstructing the TM, we recently developed a new glaucoma model based on thwarting aqueous flow to the anterior chamber by intracameral injection of silicone oil (SO), which causes pupillary block and accumulation of aqueous in the posterior chamber^20,21^. We named this SO-induced ocular hypertension model the SOHU model; it faithfully replicates the well-documented, SO-induced human secondary glaucoma that occurs as a complication of post-vitreoretinal surgery^22,23^. The SOHU model has some unique favorable features: a single SO injection produces a stable IOP elevation for a long period of time with progressive RGC and ON degeneration, but, very importantly, the ocular hypertension can be reversed quickly by SO removal^20,21,24^.

Another unique feature of the SOHU model is that, when the pupil is sufficiently dilated that it is no longer covered by the SO droplet, the anterior and posterior chambers are reconnected, allowing the aqueous to flood into the anterior chamber and be cleared away through the TM. Thus, pupil dilation removes the aqueous accumulation at the back of the eye and relieves the high IOP. We reasoned that modulating the frequency of pupil dilation in the SOHU model might change the dynamics of IOP elevation and generate different patterns of ocular hypertension-induced glaucomatous neurodegeneration that would mimic different clinical glaucoma scenarios. These modifications of the SOHU may offer advantages for understanding the mechanisms of pathogenesis and facilitating preclinical screening of neuroprotectants for different kinds of human glaucoma. Here we report three different variations of the SOHU model: (1) A chronic glaucoma model to mimic open angle glaucoma. It is generated by high frequency (HF) pupil dilation and shows sustained low IOP elevation with corresponding slow, mild glaucomatous neurodegeneration. (2) An acute high IOP elevation and severe retina degeneration model to mimic angle-closure glaucoma. It is generated by no dilation (ND) after SO injection. (3) A low frequency (LF) pupil dilation model, which we previously reported as the original SOHU model, with a phenotype that is intermediate between the two extreme models that we have developed.

## Materials and methods

### Experimental protocols statement

All experimental procedures were performed in compliance with animal protocols approved by the IACUC at Stanford University School of Medicine.

### Experimental methods statement

All experimental methods were carried out in accordance with relevant guidelines and regulations.

### Guideline statement

All experimental procedures were performed in compliance with the ARRIVE guidelines.

### Mice

C57BL/6J WT male and female mice (9–10 weeks old) were purchased from Jackson Laboratories (Bar Harbor, Maine) and maintained in a 12:12 h light–dark cycle. Control group of mice were untreated WT mice of the same age as the experimental groups. For all surgical and treatment comparisons, control and treatment groups were prepared together in single cohorts, and the experiment was repeated at least twice.

### Induction of IOP elevation by intracameral injection of SO

The detailed procedure has been published before^20,21^. In brief, mice were anesthetized by an intraperitoneal injection of Avertin (0.3mg/g) and received the SO (Alcon Laboratories, 1000 mPa.s) injection at 9–10 weeks of age. Prior to injection, one drop of 0.5% proparacaine hydrochloride (Akorn, Somerset, New Jersey) was applied to the cornea to reduce its sensitivity during the procedure. A 32G needle was tunneled through the layers of the cornea at the superotemporal side close to the limbus to reach the anterior chamber without injuring lens or iris. Following this entry, ~ 2µl silicone oil (1000 mPa s, Silikon, Alcon Laboratories, Fort Worth, Texas) was injected slowly into the anterior chamber using a homemade sterile glass micropipette, until the oil droplet expanded to cover most areas of the iris (diameter ~ 1.8–2.2mm). After the injection, veterinary antibiotic ointment (BNP ophthalmic ointment, Vetropolycin, Dechra, Overland Park, Kansas) was applied to the surface of the injected eye. The contralateral control eyes received 2 µl normal saline to the anterior chamber. During the whole procedure, artificial tears (Systane Ultra Lubricant Eye Drops, Alcon Laboratories, Fort Worth, Texas) were applied to keep the cornea moist.

### Removing SO from the anterior chamber

The detailed procedure has been published before^20,21^. Briefly, the oil droplet was removed from the anterior chamber at 2wpi. After mice were anesthetized by intraperitoneal injection of Avertin (0.3 mg/g), two corneal tunnel incisions were made using a 32G needle at the edge of the oil droplet. A 33G needle attached to an elevated balanced salt solution plus (BSS Plus, Alcon Laboratories, Ft. Worth, Texas) drip (110 cm H_2_O height, equal to 81 mmHg) was inserted through one tunnel to flow BSS into the anterior chamber to maintain its volume. At the same time, another 33G needle attached to a 1 mL syringe with the plunger removed was inserted through the other tunnel to release the SO from the anterior chamber. After removing the oil, a small air bubble was injected by a glass micropipette into the anterior chamber to maintain its volume and temporarily seal the corneal incision.

### IOP measurement/pupil dilation

Since IOP measurement requires pupil dilation, each IOP measurement can be considered a pupil dilation treatment. The detailed procedure has been published before^20,21^. Briefly, the IOP of both eyes was measured by the TonoLab tonometer (Colonial Medical Supply, Espoo, Finland) according to product instructions*.* Briefly, in the morning, mice were anesthetized with a sustained flow of isoflurane (3% isoflurane at 2 L/minute mixed with oxygen) delivered to the nose by a special rodent nose cone (Xenotec, Inc., Rolla, Missouri), which left the eyes exposed for IOP measurement. 1% Tropicamide sterile ophthalmic solution (Akorn, Somerset, New Jersey) was applied three times at 3-min intervals to fully dilate the pupils (about 10 min) before taking measurements. The average of six measurements by the TonoLab was considered as one machine-generated reading and three machine-generated readings were obtained from each eye; the mean was calculated to determine the IOP. During this procedure, artificial tears were applied to keep the cornea moist.

### Immunohistochemistry of whole-mount retina and RGC counting

The detailed procedure has been published before^20,21,25^. Briefly, after transcardiac perfusion with 4% PFA in PBS, the eyes were dissected out, post-fixed with 4% PFA for 2 h, at room temperature, and cryoprotected in 30% sucrose at 4 °C overnight. Retinas were dissected out and washed extensively in PBS before blocking in staining buffer (10% normal goat serum, Sigma-Aldrich, and 2% Triton X-100 in PBS) for half an hour. RBPMS guinea pig antibody made at ProSci Inc (Poway, California) according to publications^26,27^ was diluted (1:4000) in the same staining buffer. Floating retinas were incubated with primary antibodies overnight at 4 °C and washed 3 times for 30 min each with PBS. Secondary antibodies (Cy3) were then applied (1:200; Jackson ImmunoResearch, West Grove, Pennsylvania) and incubated for 1 h at room temperature. Retinas were again washed 3 times for 30 min each with PBS before a cover slip was attached with Fluoromount-G (SouthernBiotech, Birmingham, Alabama). For peripheral RGC counting, whole-mount retinas were immunostained with the RBPMS antibody, 8 fields sampled from peripheral regions of each retina using a 40× lens with a Zeiss M2 epifluorescence microscope, and RBPMS^+^ RGCs counted by Volocity software (Quorum Technologies) according to the following protocol: First, positive objects were identified based on a fluorescence intensity greater than the baseline intensity threshold. Next, touching objects were separated by an object size guide that excluded objects significantly smaller or larger than the normal sizes of RGCs. Before the software determined the final object count, we checked the samples and manually corrected some obvious errors. The percentage of RGC survival was calculated as the ratio of surviving RGC numbers in injured eyes compared to contralateral uninjured eyes. The investigators who counted the cells were masked to the treatment of the samples.

### ON semi-thin sections and quantification of surviving axons

The detailed procedure has been published before^20,21,25^. Briefly, after mice were perfused through the heart with ice cold 4% paraformaldehyde (PFA) in PBS, the ON was exposed by removing the brain and post-fixed in situ using 2% glutaraldehyde/2% PFA in 0.1 M PB for 4 h on ice. Samples were then washed with 0.1 M PB 3 times, 10 min each wash. The ONs were then carefully dissected out and rinsed with 0.1 M PB 3 times, 10 min each wash. They were then incubated in 1% osmium tetroxide in 0.1 M PB for 1 h at room temperature followed by washing with 0.1 M PB for 10 min and water for 5 min. ONs were next dehydrated through graded ethanol, infiltrated in propylene oxide and epoxy, and embedded in epoxy at 60 °C for 24 h. Semi-thin sections (1 µm) were cut on an ultramicrotome (EM UC7, Leica) and collected 2 mm distal to the eye. The semi-thin sections were attached to glass slides and stained with 1% para-phenylenediamine (PPD) in methanol: isopropanol (1:1) for 35 min. After rinsing 3 times with methanol: isopropanol (1:1), coverslips were applied with Permount Mounting Medium (Electron Microscopy Sciences, Hatfield, Pennsylvania). PPD stains all myelin sheaths, but darkly stains the axoplasm only of degenerating axons, which allows us to differentiate surviving axons from degenerating axons^17^. Four sections of each ON were imaged through a 100× lens of a Zeiss M2 epifluorescence microscope to cover the entire area of the ON without overlap. Two areas of 21.4 µm × 29.1 µm were cropped from the center of each image, and the surviving axons within the designated areas counted manually. After counting all the images taken from a single nerve, the mean of the surviving axon number was calculated for each ON. The mean of the surviving axon number in the injured ON was compared to that in the contralateral control ON to yield a percentage of axon survival value. The investigators who counted the axons were masked to the treatment of the samples.

### Retina ultrathin cross sections and toluidine blue staining

The detailed procedure has been published before^20,21,25^. Briefly, after mice were perfused with ice cold 4% PFA in PBS, both eyes of each animal were enucleated and fixed in 1% PFA and 1.25% glutaraldehyde fixative prepared in 0.1 mM sodium cacodylate buffer with 5 mM calcium chloride and 5% sucrose for 24 h at room temperature. Globes were then dehydrated through a graded series of alcohols, infiltrated in propylene oxide, and embedded in epoxy. Sections 0.7micron in thickness were taken using an ultramicrotome (Reichert Ultracut E; Leica, Deerfield, Illinois) and stained with 0.5% toluidine blue. Slides were photographed by a light microscope (Eclipse E1000; Nikon, Tokyo, Japan). 20× images were used to measure the thickness of the inner and outer retina at the same distance from the optic nerve head. The mean of the inner or outer retinal thickness in the injured retina was compared to that in the contralateral control retina to yield a percentage of inner or outer retinal thickness value. The investigators who measured the thickness of inner or outer retina were masked to the treatment of the samples.

### Spectral-domain optical coherence tomography (SD-OCT) imaging

The detailed procedure has been published before^20,21,25^. Briefly, after the mice were anesthetized, pupils were dilated by applying 1% tropicamide sterile ophthalmic solution (Akorn, Somerset, New Jersey), and a customized + 10D contact lens (3.0 mm diameter, 1.6 mm BC, PMMA clear, Advanced Vision Technologies) applied to the dilated pupil. The retina fundus images were captured with the Heidelberg Spectralis SLO/OCT system (Heidelberg Engineering, Germany) equipped with an 870 nm infrared wavelength light source and a 30° lens (Heidelberg Engineering). The OCT scanner has 7 µm optical axial resolution, 3.5 µm digital resolution, and 1.8 mm scan depth at 40 kHz scan rate. The mouse retina was scanned with the ring scan mode centered by the optic nerve head at 100 frames average under high-resolution mode (each B-scan consisted of 1536 A scans). The GCC includes retinal nerve fiber layer (RNFL), ganglion cell layer (GCL) and inner plexiform layer (IPL). The average thickness of GCC around the optic nerve head was measured manually with the aid of Heidelberg software. The mean of the GCC thickness in the injured retina was compared to that in the contralateral control retina to yield a percentage of GCC thickness value. The investigators who measured the thickness of GCC were masked to the treatment of the samples.

### Pattern electroretinogram (PERG) recording

PERG were recorded from both eyes simultaneously with the Miami PERG system (Intelligent Hearing Systems, Miami, Florida) according to a published protocol^28^. The procedure has been published in detail before^20,21,25^. Briefly, mice were anesthetized by xylazine and ketamine based on their body weight (0.01 mg xylazine/g + 0.08 mg ketamine/g) and placed on a feedback-controlled heating pad (TCAT-2LV, Physitemp Instruments Inc., Clifton, New Jersey) that maintained core temperature at 37 °C. A small lubricant eye drop (Systane) was applied before recording to prevent corneal dryness. The reference electrode was placed subcutaneously on the back of the head between the two ears and the ground electrode was placed at the root of the tail. The active steel needle electrode was placed subcutaneously on the snout for the simultaneous acquisition of left and right eye responses. Two 14 cm × 14 cm LED-based stimulators were placed in front of the mouse so that the center of each screen was 10 cm from each eye. The pattern remained at a contrast of 85% and a luminance of 800 cd/m^2^, and consisted of four cycles of black-gray elements, with a spatial frequency of 0.052 c/d. Upon stimulation, the independent PERG signals were recorded from the snout and simultaneously by asynchronous binocular acquisition. Two consecutive recordings of 200 traces were averaged to achieve one readout; each trace recorded up to 1020 ms. The first positive peak in the waveform was designated as P1 and the second negative peak as N2. P1 was typically around 100 ms. The amplitude was measured from P1 to N2. The mean of the P1-N2 amplitude in the injured eye was compared to that in the contralateral control eye to yield a percentage of amplitude change.

### Fundus fluorescein angiography (FFA)

After the mice were anesthetized, pupils were dilated by applying 1% tropicamide sterile ophthalmic solution (Akorn, Somerset, New Jersey), and a customized + 10D contact lens (3.0 mm diameter, 1.6 mm BC, PMMA clear, Advanced Vision Technologies) applied to the dilated pupil. 5% fluorescein sodium (3 ml/kg, AK-FLUOR, USA) were injected intraperitoneally and fundus fluorescent images, centered on the optic nerve head, were recorded from 6 s to 30 min after injection with the Heidelberg Spectralis SLO/OCT system (Heidelberg Engineering, Germany) and a 55° lens (Heidelberg Engineering).

### Statistical analyses

GraphPad Prism 7 was used to generate graphs and for statistical analyses. Data are presented as means ± s.e.m. Student’s t-test was used for two groups comparison and One-way ANOVA with post hoc test was used for multiple comparisons.

## Results

### Changing the frequency of pupil dilation after intracameral SO injection achieves three distinct patterns of IOP elevation

**Figure 1:**
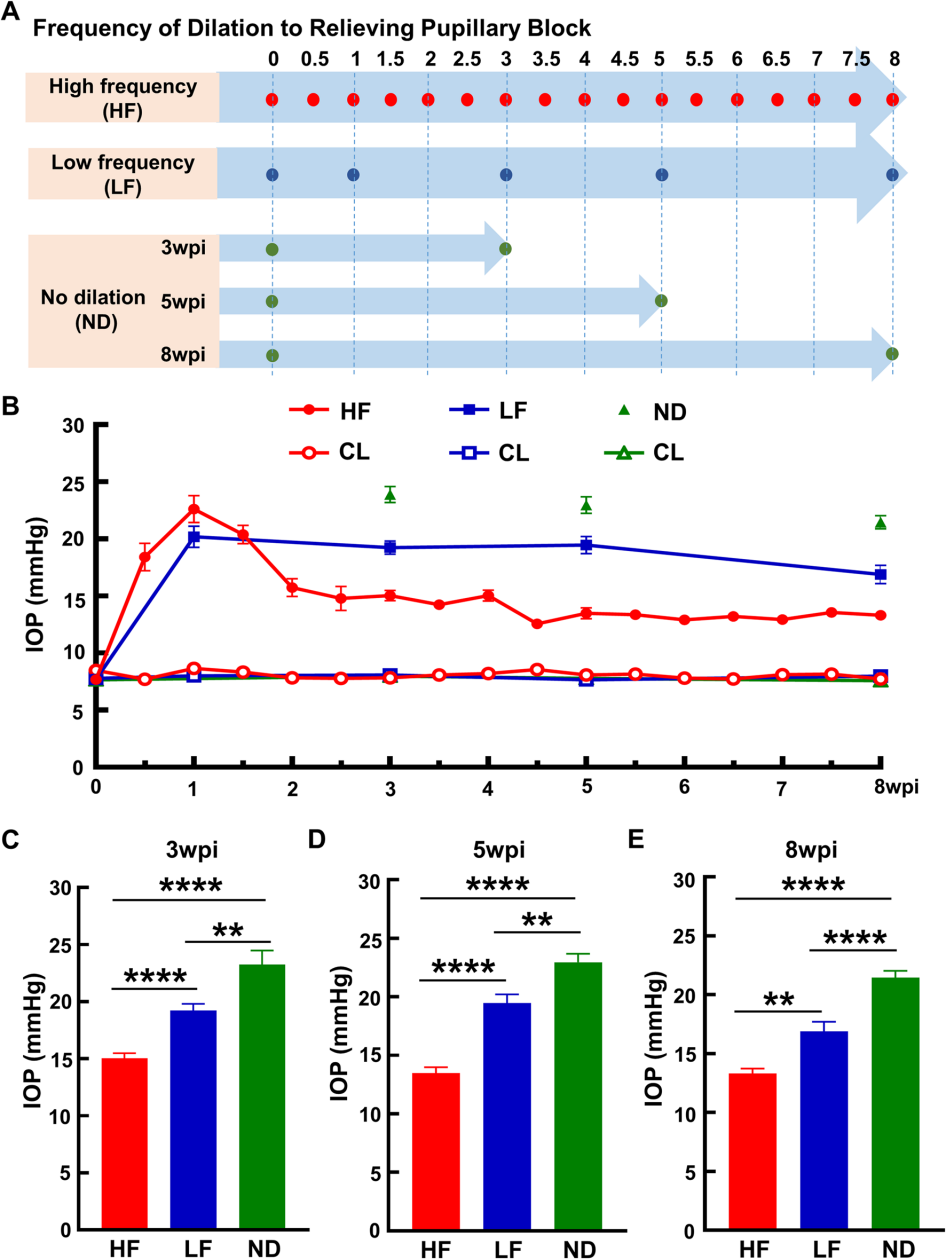
Low to high IOP elevations achieved by high to low frequencies of pupillary dilation after SO-induced ocular hypertension. (**A**) Schedule of dilation to relieve pupillary blocking after SO intracameral injection. HF: high frequency, twice a week; LF: low frequency, four times in eight weeks; ND: no dilation. wpi: weeks post SO injection. (**B**) Longitudinal IOP measurements of experimental eyes and contralateral control (CL) eyes at different time points after SO injection. ND groups at 3, 5 and 8wpi are independent groups that are sacrificed immediately after IOP measurement. (**C,D,E**) Comparisons of average IOP of the three groups at 3, 5 and 8wpi. Data are presented as means ± s.e.m, 3wpi: HF: n = 26, LF: n = 28, ND: n = 24; 5wpi: HF: n = 26, LF: n = 27, ND: n = 24; 8wpi: HF: n = 21, LF: n = 30, ND = 30. **: *p* < 0.01, ****: *p* < 0.0001, one-way ANOVA with Tukey’s multiple comparison test.

We previously developed a simple, inducible and reversible mouse ocular hypertension model based on anterior chamber injection of SO. This model faithfully mimics secondary glaucoma seen in the clinic, including pupillary blocking that prevents inflow of aqueous into the anterior chamber, significant IOP elevation in the posterior segment, and severe RGC loss and ON degeneration^20,21^. We confirmed that when the size of the dilated pupil exceeds that of the SO droplet or when the SO droplet is removed from the eye, aqueous will migrate into the anterior chamber and relieve the IOP elevation^20,21^. We reasoned that relieving pupillary blocking by pupil dilation at different frequencies would generate different IOP elevation patterns that would mimic different types of glaucoma patients. As illustrated in Fig. 1A, we tested three pupil dilation schemes after SO injection. In addition to the original SOHU model, in which the pupil is dilated 4 times in 8 weeks and which we term LF here, we tested another two schemes: HF (twice a week for 8 weeks) and no dilation (ND). We measured IOP each time that we dilated the pupils. Interestingly, although all the SO-injected eyes had significantly higher IOP than the contralateral control (CL) eyes, these three dilation conditions generated three distinct patterns of IOP elevation (Fig. 1B–E): lowest in the HF group, highest in the ND group, intermediate in the LF group. These dilation conditions therefore allow us to model the clinical scenarios of patients with moderate to high IOP elevations.

### Mild to severe RGC and ON degeneration correlates with moderate to high IOP elevation in the HF, LF and ND groups

**Figure 2:**
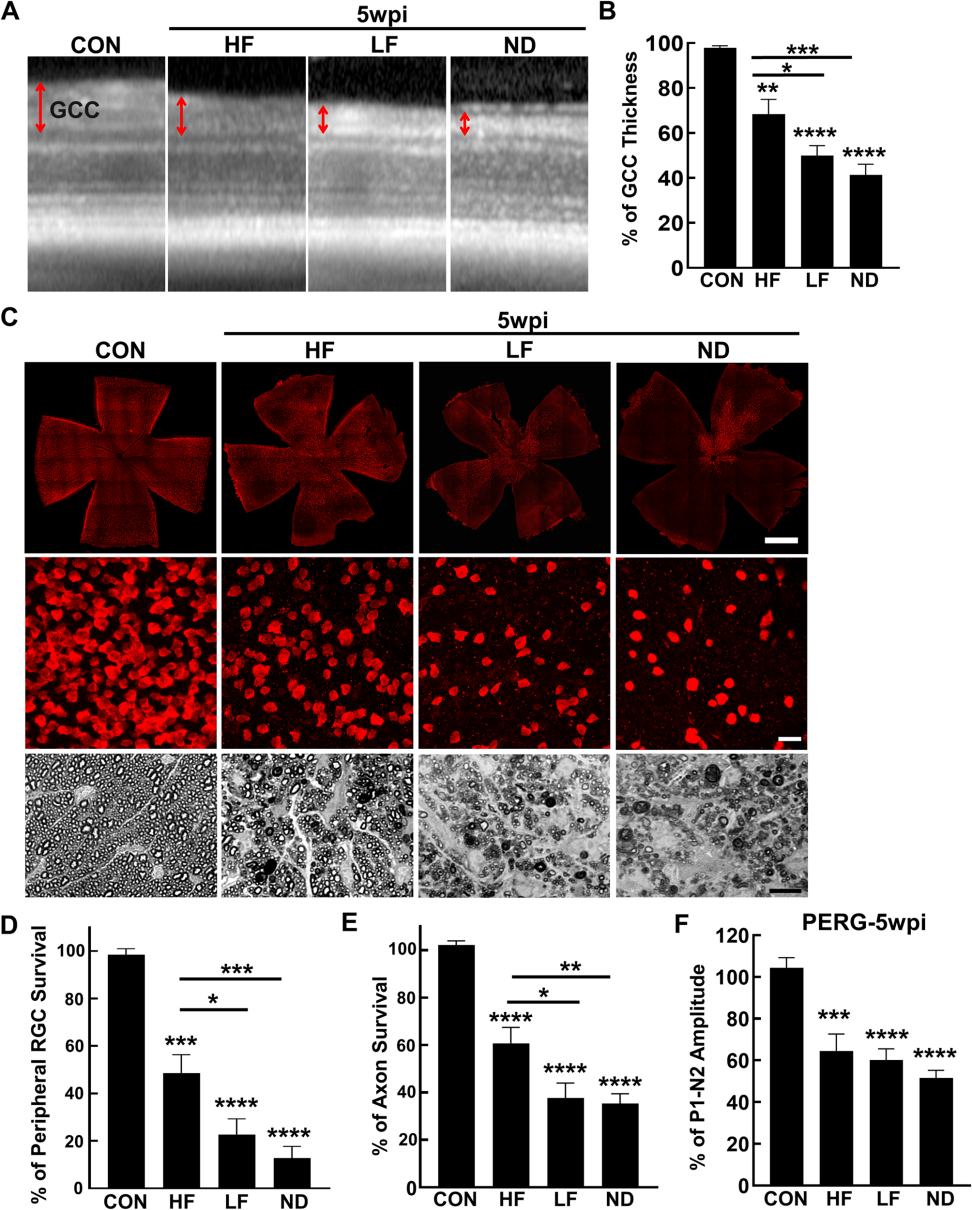
Mild to severe glaucomatous RGC soma and axon degeneration in HF, LF and ND eyes at 5wpi. (**A**) Representative OCT images of mouse retinal area surrounding the ON head at 5wpi. GCC indicated by double end arrows. (**B**) Quantification of GCC thickness, represented as percentage of GCC thickness in the SO eyes, compared to the CL eyes. Control (CON): n = 16; HF-5w: n = 25; LF-5w: n = 27; ND-5w: n = 22; ND-3w: n = 24, * *P* < 0.05, ***: *p* < 0.001, ****: *p* < 0.0001, one-way ANOVA with Tukey’s multiple comparison test. (**C**) Upper panel, confocal images of wholemount retinas showing surviving RBPMS-positive (red) RGCs of naïve, HF, LF and ND eyes at 5wpi. Scale bar, 100 µm. Middle panel, confocal images of a portion of the peripheral retina showing surviving RBPMS-positive (red) RGCs in the corresponding groups. Scale bar, 20 µm. Lower panel, light microscope images of semi-thin transverse sections of ON stained with PPD in the corresponding groups. Scale bar, 10 µm. (**D,E**) Quantification of surviving RGCs in the peripheral retina and surviving axons in ON of the corresponding groups at 5wpi, represented as percentage of SO eyes compared to CL eyes. (**F**) Quantification of P1-N2 amplitude, represented as percentage of P1-N2 amplitude in the SO eyes, compared to CL eyes. Data are presented as means ± s.e.m, Control: n = 12; HF: n = 22; LF: n = 16; ND-5w: n = 23; * *P* < 0.05, ** *P* < 0.01, ***: *P* < 0.001, ****: *P* < 0.0001; one-way ANOVA with Tukey’s multiple comparison test.

**Figure 3:**
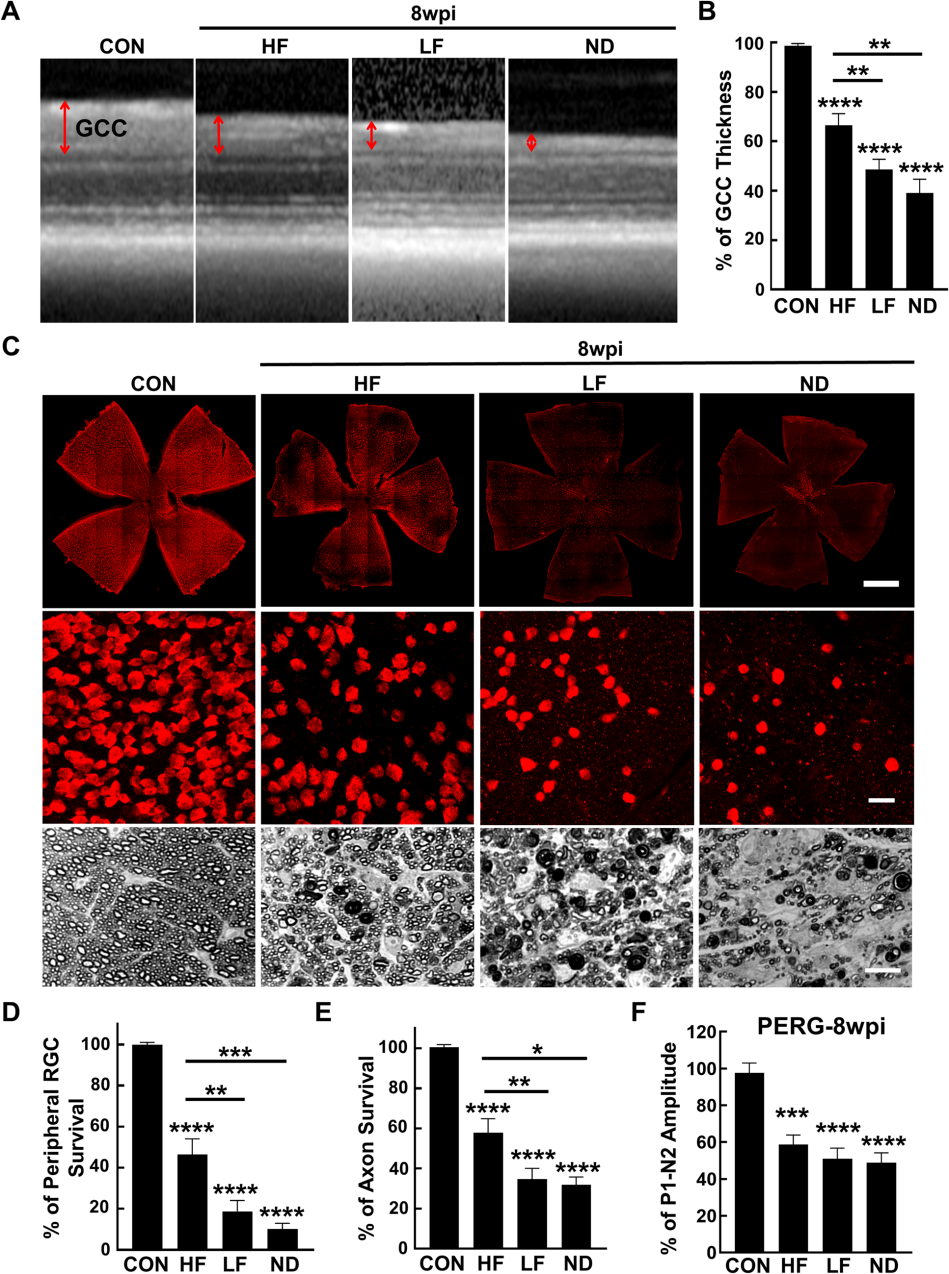
Mild to severe glaucomatous RGC soma and axon degeneration in HF, LF and ND eyes at 8wpi. (**A**) Representative OCT images of mouse retinal area surrounding the ON head. GCC: ganglion cell complex, including RNFL, GCL and IPL layers; indicated by double end arrows. (**B**) Quantification of GCC thickness, represented as percentage of GCC thickness in the SO eyes, compared to the CL eyes. Control (CON): n = 17; HF: n = 30; LF: n = 30; ND: n = 14. **: *p* < 0.01, ****: *p* < 0.0001, one-way ANOVA with Tukey’s multiple comparison test. (**C**) Upper panel, confocal images of wholemount retinas showing surviving RBPMS-positive (red) RGCs of naïve, HF, LF and ND eyes at 8wpi. Scale bar, 100 µm. Middle panel, confocal images of a portion of the peripheral retina showing surviving RBPMS-positive (red) RGCs in the corresponding groups. Scale bar, 20 µm. Lower panel, light microscope images of semi-thin transverse sections of ON stained with PPD in the corresponding groups. Scale bar, 10 µm. (**D,E**) Quantification of surviving RGCs in the peripheral retina and surviving axons in ON of the corresponding groups at 8wpi, represented as percentage of SO eyes compared to CL eyes. (**F**) Quantification of P1-N2 amplitude, represented as percentage of P1-N2 amplitude in the SO eyes, compared to CL eyes. Data are presented as means ± s.e.m. Control (CON): n = 12; HF: n = 26; LF: n = 30; ND: n = 27, * *p* < 0.05, ** *p* < 0.01, ***: *p* < 0.001, ****: *p* < 0.0001; one-way ANOVA with Tukey’s multiple comparison test.

To compare the glaucomatous neurodegeneration in these three different IOP elevation scenarios, we first used OCT to measure the ganglion cell complex (GCC) thickness in live animals at two time points, 5wpi and 8wpi. The GCC includes retina nerve fiber layer (RNFL), ganglion cell layer (GCL) and inner plexiform layer (IPL), which reflects the integrity of RGC axons, somata, and dendrites^20,21,25,29,30^. At both these time points, GCC became significantly thinner than control group in all three groups of SOHU eyes, but was significantly thicker in the HF group than in the other two groups (Figs. 2A,B, 3A,B). These results indicate that the moderate to high IOP elevations in the HF, LF and ND groups correlate with mild to severe glaucomatous neurodegeneration.

Histological analysis of post-mortem retina and ON consistently supported the OCT findings. We quantified surviving RGC somata in retinal wholemounts and surviving axons in ON semithin cross-sections at 5wpi and 8wpi. Similar to the changes of GCC thickness measured by OCT in vivo, there was significant and worsening RGC somata loss in the HF, LF and ND groups at these two time points (Figs. 2C,D, 3C,D). Significant and worsening RGC axon degeneration also occurred in the HF, LF and ND groups (Figs. 2C,E, 3C,E). Therefore, moderate IOP elevation in the HF group produces significant but mild glaucomatous RGC and ON degeneration, whereas higher IOP elevations in the LF and ND groups cause significantly more severe RGC and ON degeneration.

Pattern electroretinogram (PERG) offers an important electrophysiological assessment of general RGC function; the ERG responses are stimulated with contrast-reversing horizontal bars alternating at constant mean luminance^31^. Our PERG system measured both eyes at the same time, which provides an internal control for reference and normalization to minimize variations. Consistent with RGC morphological deficits, the P1-N2 amplitude ratio of the SO eyes to CL eyes decreased significantly in the three groups (Figs. 2F, 3F).

### Inner retina degeneration is dramatically more severe in the ND group than in the HF and LF groups

**Figure 4:**
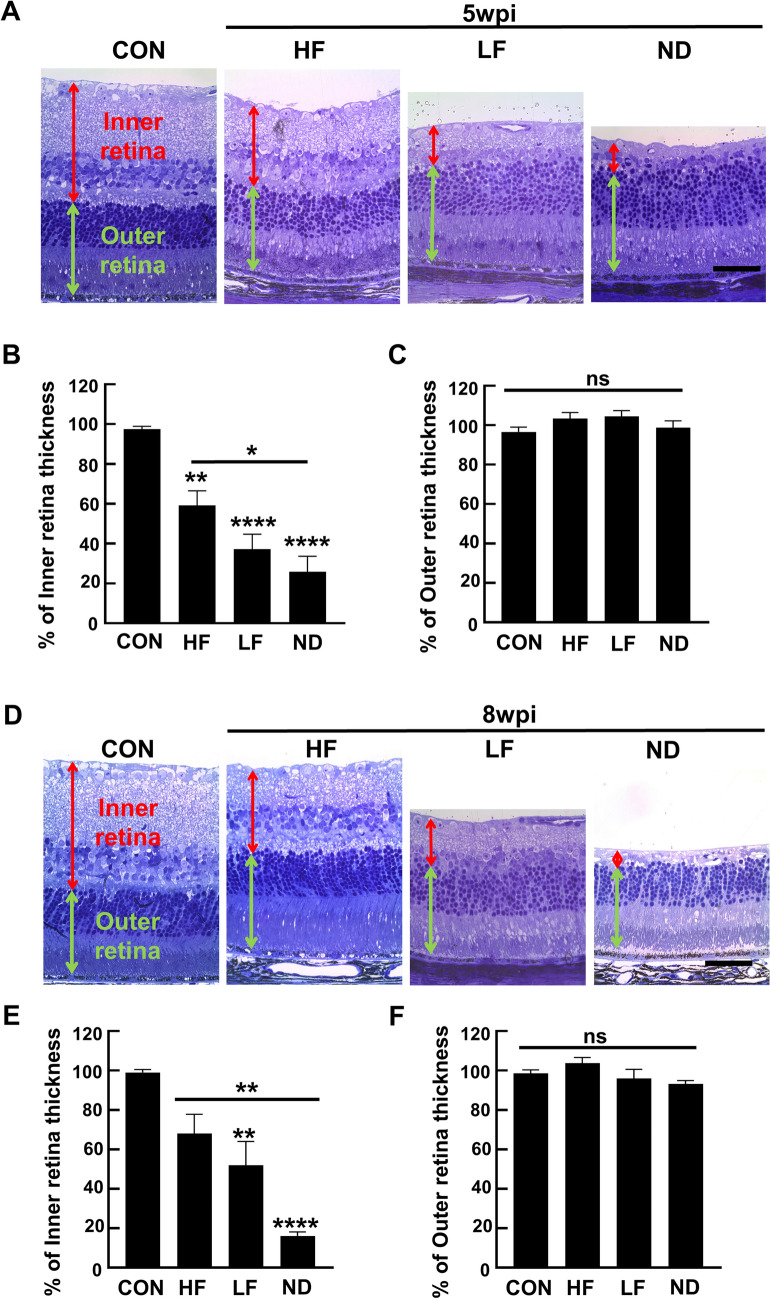
Degeneration of the inner retina: severe in ND, but much milder in HF and LF groups. (**A,D**) Representative light microscope images of semi-thin transverse sections of retina stained with toluidine blue in the corresponding groups at 5 and 8wpi. Scale bar, 50 µm. (**B,E**) Quantification of inner retina thickness at 5 and 8wpi, represented as percentage of SO eyes compared to CL eyes. (**C,F**) Quantification of outer retina thickness at 5 and 8wpi, represented as percentage of SO eyes compared to CL eyes. Data are presented as means ± s.e.m. 5wpi: Control (CON): n = 8; HF: n = 10; LF: n = 10; ND: n = 10; 8wpi: Control (CON): n = 8; HF: n = 10; LF: n = 10; ND: n = 6, * *p* < 0.05, ** *p* < 0.01; one-way ANOVA with Tukey’s multiple comparison test.

Since high IOP elevation may reduce blood flow to the retina and cause ischemic damage^32–35^, we used histological analysis of retinal cross-sections to further characterize the changes of the entire retina in the three scenarios of SOHU eyes. In dramatic contrast to the HF and LF groups, we found that the inner retina of the ND group was significantly degenerated at 5wpi (Fig. 4A,B) and that the degeneration had worsened at 8wpi, the entire inner retina of the ND groups had almost completely disappeared at 8wpi (Fig. 4D,E); whereas there was no significant outer retina degeneration (Fig. 4C,F).

**Figure 5:**
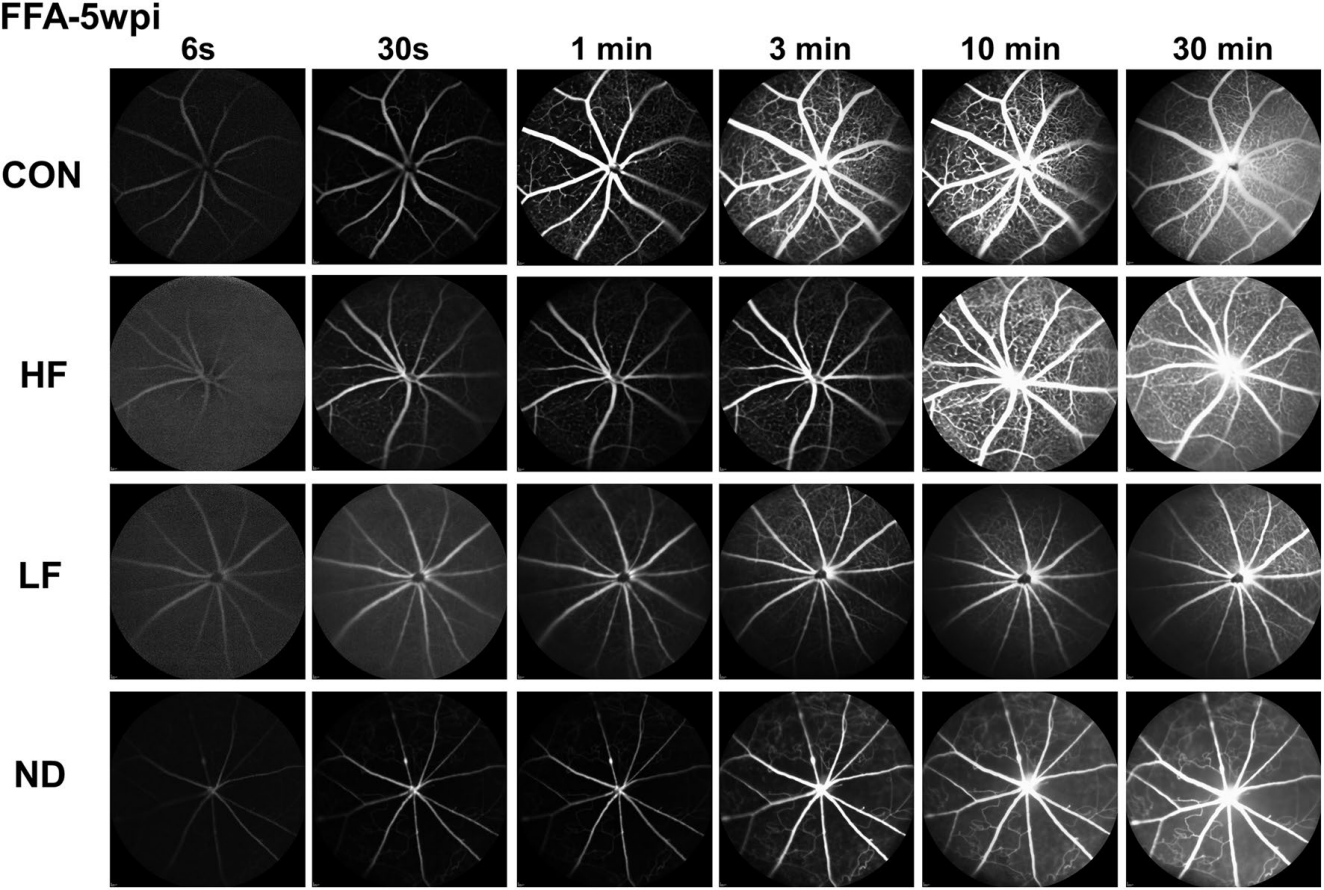
Decreased blood supply in the inner retina: severe in ND, much milder in HF and LF groups**.** Time series of retinal fundus fluorescein angiography (FFA) images at different time points after fluorescein injection show significantly delayed retinal vessel filling and marked capillary non-perfusion in the ND group, whereas the HF group is similar to the control (CON) group, at 5wpi. Three animals of each group were imaged.

To determine whether ischemia causes the severe inner retina degeneration in the ND group, we investigated flow in the inner retina blood vessels using fundus fluorescein angiography (FFA)^36–38^. We consistently found that blood flow was significantly delayed and decreased in ND retinas and to a lesser degree in LF retinas, whereas blood infiltration dynamics in HF retinas were similar to those in control retinas (Fig. 5). Moreover, severe capillary non-perfusion was observed in the ND group, while there was no obvious non-perfusion area in the HF group (Fig. 5).

### Removal of SO and reversal of ocular hypertension do not stop the progression of glaucomatous neurodegeneration in the HF group

**Figure 6:**
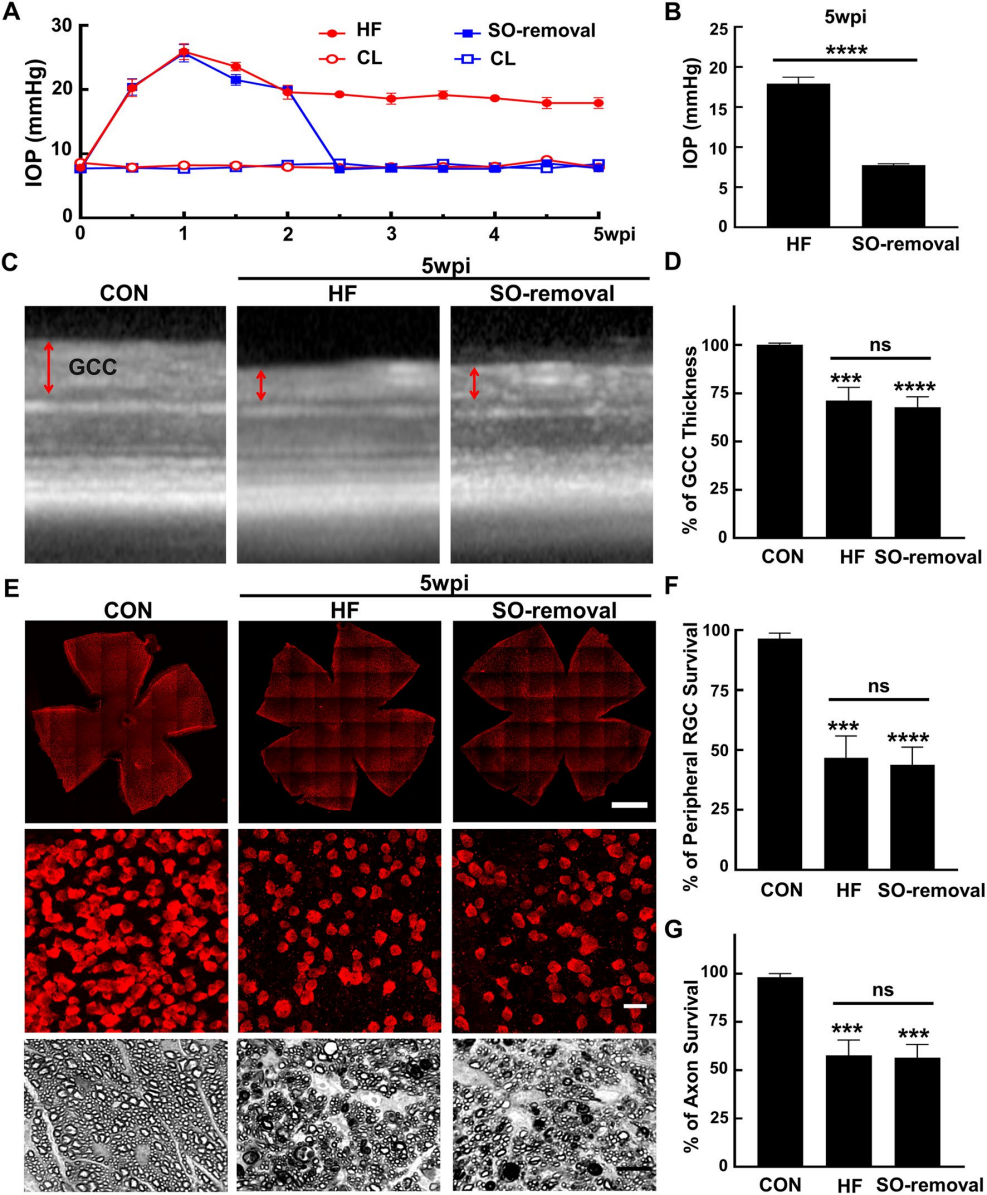
SO removal reverses IOP elevation but not glaucomatous neurodegeneration in HF group. (**A**) Longitudinal IOP measurements of HF SOHU eyes before and after SO removal and CL eyes at different time points after SO injection. (**B**) Comparisons of average IOP of the SOHU eyes with or without SO-removal at 5wpi. Data are presented as means ± s.e.m, n = 12. ****: *p* < 0.0001, Student’s t test. (**C**) Representative OCT images of mouse retina area surrounding ON head at 5wpi. GCC indicated by double end arrows. (**D**) Quantification of GCC thickness, represented as percentage of GCC thickness in the SO eyes, compared to the CL eyes. Control (CON): n = 13; HF: n = 19; SO-removal: n = 19, ****: *p* < 0.0001, one-way ANOVA with Tukey’s multiple comparison test. (**E**) Upper panel, confocal images of wholemount retinas showing surviving RBPMS-positive (red) RGCs of naïve, HF, and SO removal eyes at 5wpi. Scale bar, 100 µm. Middle panel, confocal images of a portion of the peripheral retina showing surviving RBPMS-positive (red) RGCs in the corresponding groups. Scale bar, 20 µm. Lower panel, light microscope images of semi-thin transverse sections of ON stained with PPD from the corresponding groups. Scale bar, 10 µm. (**F,G**) Quantification of surviving RGCs in the peripheral retina and surviving axons in ON of the corresponding groups at 5wpi, represented as percentage of SO eyes compared to CL eyes. Data are presented as means ± s.e.m, Control (CON): n = 12; HF: n = 22; SO-Removal: n = 22, ***: *p* < 0.001, ****: *p* < 0.0001; one-way ANOVA with Tukey’s multiple comparison test.

One of the advantages of the SOHU model is that the cause of the pathology, SO-induced pupillary blocking and IOP elevation, is reversible. We next asked whether reversing ocular hypertension, which mimics treatment by IOP reduction in the clinic, affects the progression of glaucomatous neurodegeneration in the HF group. Consistent with our previous findings^20,21^, we were able to flush out the SO from the anterior chamber and lower the IOP to normal quickly and stably at 2wpi (Fig. 6A,B). Interestingly, although the ocular hypertension was reversed in the SO-removal HF group for 3 weeks, the GCC thinning and RGC soma and axon loss were indistinguishable from the HF group without SO removal at 5wpi (Fig. 6C–G). This result is strikingly similar to the clinical observation that lowering the IOP can delay but not prevent glaucomatous neurodegeneration, at least in some patients. It indicates that the initial IOP elevation is pathogenic and that IOP elevation for two weeks suffices to cause progressive glaucomatous neurodegeneration.

## Discussion

By a simple procedure, varying the frequency of pupillary dilation (Fig. 1A), we extend our original SOHU model into two additional clinically relevant glaucoma models: (1) a chronic but mild glaucomatous neurodegeneration HF model with stable, moderate IOP elevation that mimics human chronic open angle glaucoma; (2) an acute, severe glaucomatous neurodegeneration ND model with sustained high IOP elevation that accurately recapitulates human acute angle-closure glaucoma. Primary open angle glaucoma is three times more common than angle-closure glaucoma, and is normally associated with a gradual increase in IOP and slowly progressive RGC and ON loss^39^. In the HF model, we follow the SO injection by dilating the pupil twice weekly to frequently relieve the aqueous accumulation in the posterior of the eye. As in chronic open angle glaucoma, this procedure elevates IOP to a moderate level (Fig. 1B–E) and induces mild but significant RGC and ON degeneration at 5 and 8wpi (Figs. 2,3), without obvious degeneration in other layers of the retina (Fig. 4).

In contrast, in acute angle-closure glaucoma outflow blockade of the aqueous humor increases IOP rapidly^40^. Failure to control IOP in a timely manner produces temporary or permanent RGC/ON damage and vision loss. Although angle-closure glaucoma is much less frequent than open angle glaucoma, it is responsible for 50% of blindness caused by glaucoma^8^. In the ND model, we do not dilate the pupil after SO injection. Therefore, there is no relief of pupillary block and IOP is maintained at a much higher level. The sustained high IOP elevation induces significantly more severe RGC and ON degeneration (Figs. 2–4), closely resembling the rapid, severe degeneration in angle-closure glaucoma. Even more dramatically, in the ND model the entire inner retina is degenerated (Fig. 4), suggesting ischemic damage. Ocular vascular dysfunction has long been known to be correlated with the incidence of glaucoma^32–35^. Retina has a dual blood supply system: the outer retina, including outer nuclear layer, outer plexiform layer and the majority of the ON head, is supplied by the choroidal vascular bed derived from the posterior ciliary arteries; the inner retina, in contrast, is supplied by the central retinal artery^41^. Acute high IOP elevation in patients with angle-closure glaucoma can induce central retinal artery occlusion with ischemic damage of the inner retina^32–35^. Indeed, ocular ischemia with inner retina damage and outer retina sparing has been reported in rats with acute high IOP elevation^42–45^. FFA is a diagnostic method for vascular deformation, non-perfusion and leakage, which can be used to evaluate the retinal blood supply^36–38^. It revealed significantly decreased blood flow in the inner retina of the ND group (Fig. 5), indicating that the severe retinal ischemia induced by marked ocular hypertension caused significant inner retinal atrophy in these mice. This ischemia is another similarity to the clinical syndrome and increases the relevance of the ND paradigm as a valuable acute angle-closure model.

Another surprising and interesting finding of the study is that although SO removal allows IOP to return quickly to normal, it does not stop the progression of glaucomatous neurodegeneration in the HF model. As Fig. 6 shows, when SO is removed at 2wpi from one group of mice with the HF model and the mice compared 3 weeks later to HF mice without SO removal at 5wpi, the two groups show similar RGC and ON degeneration. This result is also consistent with the clinical observation that visual field loss can progress aggressively in some glaucoma patients whose IOP is maintained at a relatively low level. It will be very interesting to determine what causes the continuing neurodegeneration after removal of the initial pathogenic high IOP. One possible explanation is that inflammatory, excitotoxic or glial responses to initial ocular hypertension are sustained in retina and ON after IOP returns to normal and continue to degrade RGCs and ON. Another possibility is that intrinsic RGC and ON degeneration signaling pathways are independent of IOP elevation after being activated. This variant of the HF model with SO removal will therefore also be useful for determining the mechanism for ocular hypertension-initiated but IOP-independent glaucomatous neurodegeneration. We did not perform the same assay in the ND model because the onset and progression of the neurodegeneration are too rapid and severe.

Glaucoma is the leading cause of irreversible blindness worldwide. To facilitate investigating the molecular mechanisms of glaucomatous degeneration and assessing the efficacy of experimental neuroprotectants, the present study has used a straightforward modification of our original SOHU model to establish two additional distinct mouse glaucoma models. We recommend using the HF model to study chronic, mild open angle glaucoma-like neurodegeneration and the clinical scenario of treatments that lower IOP; and the ND model to study acute, severe angle-closure glaucoma-like retina degeneration and ischemia. Both models will be very valuable for preclinical selection of effective neuroprotective therapies.
